# Fine particulate matter contributes to COPD-like pathophysiology: experimental evidence from rats exposed to diesel exhaust particles

**DOI:** 10.1186/s12931-023-02623-y

**Published:** 2024-01-04

**Authors:** Zhang-fu Fang, Zhao-ni Wang, Zhe Chen, Yang Peng, Yu Fu, Yang Yang, Hai-long Han, Yan-bo Teng, Wei Zhou, Damo Xu, Xiao-yu Liu, Jia-xing Xie, Junfeng (Jim) Zhang, Nan-shan Zhong

**Affiliations:** 1 Department of Respirology & Allergy, Third Affiliated Hospital of Shenzhen University, Shenzhen, 518020 China; 2 State Key Laboratory of Respiratory Disease, National Clinical Research Center for Respiratory Disease, Guangzhou Institute of Respiratory Health, The First Affiliated Hospital of Guangzhou Medical University, Guangzhou, 510120 China; 3 Laboratory of Cough, Affiliated Kunshan Hospital of Jiangsu University, Suzhou, 215300 Jiangsu China; 4 Global Health Research Center, Duke Kunshan University, Kunshan, 215316 Jiangsu Province China; 5 State Key Laboratory of Respiratory Disease Allergy Division at Shenzhen University, Institute of Allergy & Immunology, Shenzhen University School of Medicine, Shenzhen, 518061 China; 6 Nicholas School of the Environment and Global Health Institute, Duke University, Durham, NC 27708 USA; 7 Guangzhou Laboratory, Guangzhou, 510000 China

**Keywords:** PM_2.5_, Diesel engine exhaust, Small airway remodeling, Emphysema, Eosinophils, COPD

## Abstract

**Background:**

Ambient fine particulate matter (PM_2.5_) is considered a plausible contributor to the onset of chronic obstructive pulmonary disease (COPD). Mechanistic studies are needed to augment the causality of epidemiologic findings. In this study, we aimed to test the hypothesis that repeated exposure to diesel exhaust particles (DEP), a model PM_2.5_, causes COPD-like pathophysiologic alterations, consequently leading to the development of specific disease phenotypes. Sprague Dawley rats, representing healthy lungs, were randomly assigned to inhale filtered clean air or DEP at a steady-state concentration of 1.03 mg/m^3^ (mass concentration), 4 h per day, consecutively for 2, 4, and 8 weeks, respectively. Pulmonary inflammation, morphologies and function were examined.

**Results:**

Black carbon (a component of DEP) loading in bronchoalveolar lavage macrophages demonstrated a dose-dependent increase in rats following DEP exposures of different durations, indicating that DEP deposited and accumulated in the peripheral lung. Total wall areas (WAt) of small airways, but not of large airways, were significantly increased following DEP exposures, compared to those following filtered air exposures. Consistently, the expression of α-smooth muscle actin (α-SMA) in peripheral lung was elevated following DEP exposures. Fibrosis areas surrounding the small airways and content of hydroxyproline in lung tissue increased significantly following 4-week and 8-week DEP exposure as compared to the filtered air controls. In addition, goblet cell hyperplasia and mucus hypersecretions were evident in small airways following 4-week and 8-week DEP exposures. Lung resistance and total lung capacity were significantly increased following DEP exposures. Serum levels of two oxidative stress biomarkers (MDA and 8-OHdG) were significantly increased. A dramatical recruitment of eosinophils (14.0-fold increase over the control) and macrophages (3.2-fold increase) to the submucosa area of small airways was observed following DEP exposures.

**Conclusions:**

DEP exposures over the courses of 2 to 8 weeks induced COPD-like pathophysiology in rats, with characteristic small airway remodeling, mucus hypersecretion, and eosinophilic inflammation. The results provide insights on the pathophysiologic mechanisms by which PM_2.5_ exposures cause COPD especially the eosinophilic phenotype.

**Supplementary Information:**

The online version contains supplementary material available at 10.1186/s12931-023-02623-y.

## Background

Chronic obstructive pulmonary disease (COPD) has become the third leading cause of death worldwide and its prevalence is still on the rise [1]. Exposure to noxious particles and gases is considered a main cause of COPD. Among those, tobacco smoking is a confirmed and major etiology of COPD globally [2]. However, up to 45% COPD patients have never smoked, and about 26–53% of the COPD cases may be attributed to other environmental exposures [3–5]. For example, a large nationwide cross-sectional study in China found that exposure to high ambient concentrations of fine particulate matter (PM_2.5_: particulate matter with aerodynamic diameter < 2.5 μm) significantly increased the prevalence of COPD by two-fold [6]. Additionally, some large cohort studies have revealed positive associations of long-term ambient PM_2.5_ exposure and higher incidence and prevalence of COPD [7, 8]. People residing close to the urban traffic have been significantly associated with a higher risk of having COPD [9, 10]. However, the epidemiology on PM_2.5_ as an COPD etiology is still elusive. In a recent official American Thoracic Society (ATS) workshop report, ambient PM_2.5_ is considered as a plausible contributor to the onset of COPD [11]. More mechanistic studies are needed to ascertain the causal role of chronic exposure to PM_2.5_ in the development of COPD and related phenotypes.

COPD is a heterogeneous disease characterized by fixed airflow limitation. Small airway remodeling and emphysematous destruction are two main pathologic features of this disease. Two seminal studies have shown that small airways are the primary site for airflow limitation, and that dysfunction of the small airways contributed significantly to airflow limitation in patients with COPD [12, 13]. Emerging epidemiologic studies have recently shown associations between ambient air pollution, especially PM_2.5_, and small airway dysfunction. In a nationwide survey, Xiao et al. revealed that spirometry-defined small airway dysfunction was significantly associated with exposure to high level of ambient PM_2.5_ [14]. Two indoor air filtration studies reported that reduced PM_2.5_ exposure was significantly associated with improved small airway mechanics in young adults [15] and in children [16]. However, a direct link between PM_2.5_ effects on the small airway and COPD pathogenesis is yet to be established, which is difficult to do in epidemiologic studies. Hence, we designed the present experimental study to test our hypothesis that repeat exposure to ambient PM_2.5_ causes small airway remodeling as the primary site of injury, consequently leading to the development of experimental COPD.

It is known that inflammatory phenotypes play an important role in COPD pathogenesis. Cigarette smoke-related COPD is usually characterized by a neutrophilic-dominant inflammatory phenotype, while biomass smoke-related COPD can manifest as eosinophilic-biased airway inflammation [17, 18]. However, the effect of PM_2.5_ on inflammatory phenotypes of COPD remains unknown. We, hence, aim to investigate COPD phenotypes (e.g., eosinophilic versus neutrophilic) that are more likely to be induced by fine particulate matter exposure.

Diesel exhaust particles (DEP) generated by traffic vehicles are a major source of ambient PM_2.5_ in urban areas. Freshly generated DEP contains fine and ultrafine particles coated by complex hazardous components, and has been used as a model PM_2.5_ in numerous toxicological studies [19, 20]. Experimental evidence has shown that inhaled aerosol particles ranged in smaller sizes deposited mainly in the small airways of rodents [21, 22]. In this regard, DEP represents an ideal model to investigate the injury in the small airways and will be used in the present study. Using our established DEP inhalation exposure protocol for rats [23], we will evaluate COPD-relevant morphologic changes of various compartments of the airways, airway inflammation and lung function, following DEP exposures of various durations.

## Materials and methods

### Experimental animals

A total of 36 Male Sprague Dawley (SD) rats (6 weeks old with body weight around 200 g) were purchased from the Guangdong Medical Laboratory Animal Center (Foshan, China). All animals were housed in pathogen-free facilities. The animals were maintained in standard cages with food and water available *ad libitum*. Animals were divided into six groups by exposure condition, including the filtered-air controls, 2-week (wk) DEP exposure, 4-wk DEP exposure and 8-wk DEP exposure. Each group had 6 rats. The animal experimental protocols were approved by the Animal Care and Use Committee of Guangzhou Medical University and confirmed to the Guide for the Care and Use of Laboratory Animals.

### DEP exposure protocol

**Fig. 1:**
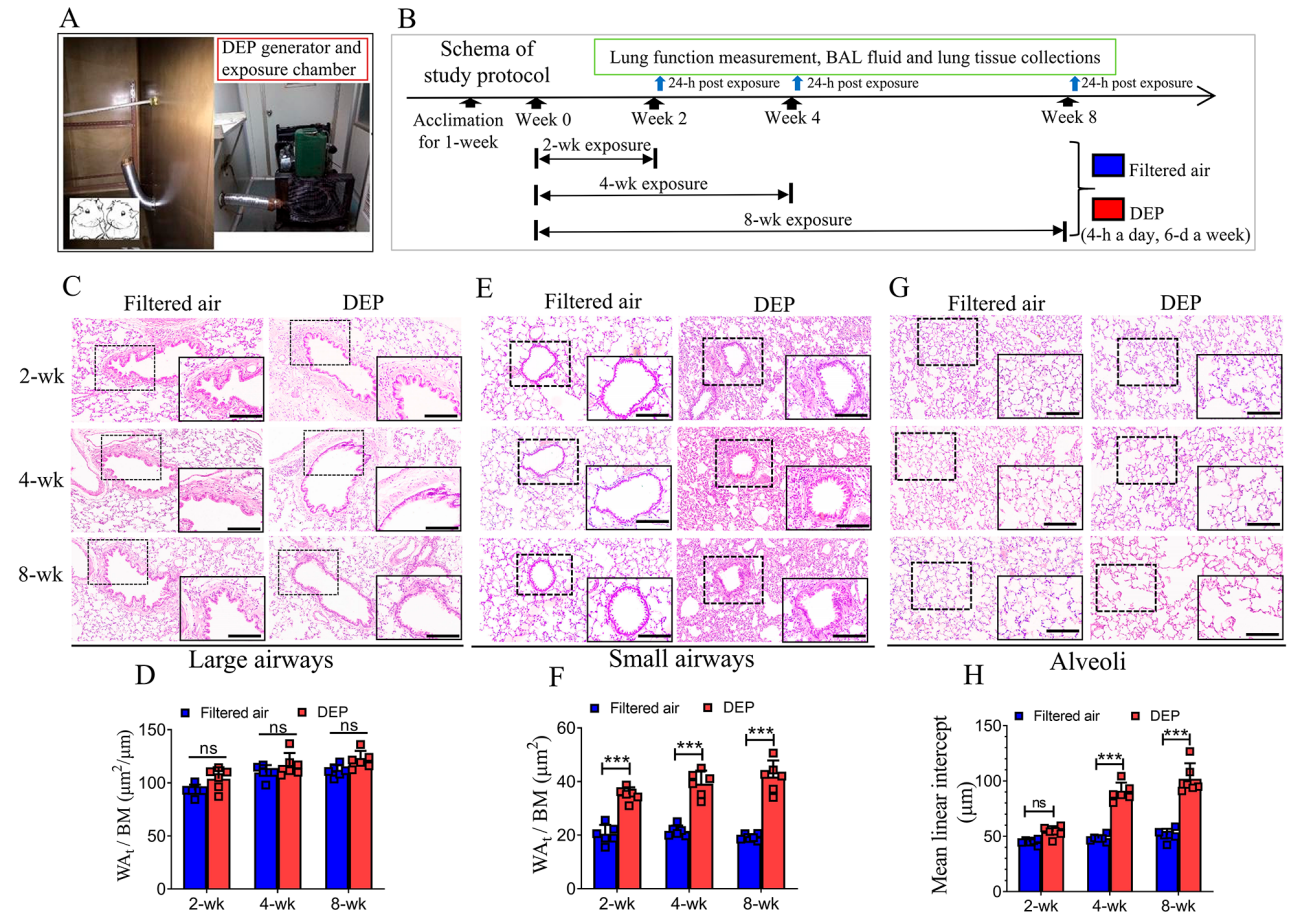
Study protocol and structural changes in different compartments of rat lung. The DEP generator and exposure chamber were shown in (**A**), while the schema of study protocol was demonstrated in (**B**). Total wall area (WAt) of large airways showing no significant difference following 2-wk, 4-wk and 8-wk DEP exposures, respectively, as compared to the filtered air controls (**C-D**). DEP exposures for three durations induced significant increases in WAt of small airways (**E-F**). Compared to the filtered air control, 4-wk and 8-wk DEP exposures, respectively, resulted in significant increases in MLI of alveoli (**G-H**). Data were expressed as mean (95% CI). ns = not significant, *** *p* < 0.001. n = 6 rats/group. Bars in all the HE staining images = 100 μm

The DEP generator and exposure chamber are shown in Fig. 1A. In brief, the exposure chamber had size dimensions of 3.3 m × 2.2 m × 2.2 m. The walls inside the exposure chamber were coated with Teflon. Filtered air was introduced into the chamber through an air pump (2.5 L/min) located above the ceiling of the room. Fresh diesel exhaust particles (DEP) were generated by a 2.0 L diesel automotive engine (Nissan, M1D, Japan), using diesel fuel (Sinopec Corp., #0, China). A small fan was running inside the tightly-enclosed chamber to mix the DEP uniformly during the experiment. An exhaust fan connected to a ventilating duct was installed to allow the rapid discharge of the gaseous pollutants. DEP concentrations were maintained at a steady-state in the chamber.

The experimental protocol can be referred to Fig. 1B. In brief, the DEP-exposed rats were placed in the chamber to inhale freshly generated DEP, 4 h a day and 6 days a week, for consecutive 2, 4, or 8 weeks, respectively. Control rats were exposed to filtered ambient air under the same chamber settings. The temperature and relative humidity inside the chamber for all experiments were controlled at 21.0 °C and 60.0%, respectively.

### Air pollutants measurements

During the exposure periods, DEP mass was collected onto quartz fiber filters (Whatman, QM-A, UK) using high-volume particulate matter samplers (Thermo, GUV-15HBL1, USA). DEP mass collected was determined gravimetrically by weighing the quartz filters before and after sampling; and particle mass concentrations (PMC) were calculated by dividing DEP mass by the air volume sampled. Particle number concentrations (PNC) of DEP were obtained by Dylos particle counting device (Dylos DC1700™, USA), of which has been validated to estimate the fine particulate matter concentration in a previous study [24]. Pollutant concentrations were measured at one-minute resolution during 4 h DEP exposures. Chemical components of DEP, e.g., organic carbon (OC), elemental carbon (EC), metal elements and polycyclic aromatic hydrocarbons (PAHs), were analyzed as described below.

### Chemical analysis of DEP

A punch (1.5 × 1.0 cm) of each filter was taken for the measurements of OC and EC using a thermal/optical transmittance aerosol carbon analyzer (Sunset Laboratory Inc., OR, USA). For metal elements, the extraction and instrumental analyses were executed according to the method described previously [25]. Briefly, four punches (1.5 × 1.0 cm) of each filter were first extracted in a digestion bomb (CEM Corp., XP-1500, USA) with a mixture of 4 ml of HNO_3_ (Merck Corp., Germany), 1 ml of HCl (Guangzhou Chemical Reagent Factory, China), and 0.2 ml of HF (Guangzhou Chemical Reagent Factory, China), and digested by a microwave digestion unit (CEM Corp., MARS 5, USA). The digestion was carried out at 175 °C for 25 min. The resulting solution was diluted to 50 ml with ultra-pure water generated from a Milli-Q system (Millipore Corp., USA). The final solutions were measured by an ICP-MS equipped with a Babington nebulizer (Agilent Corp., 7700X, USA). For measuring PAHs, filters were extracted with 30 mL dichloromethane (Merck Corp., Germany) using ultrasonic agitation under 30 °C and filtered. This extraction procedure was repeated three times. The combined extracts were filtered and concentrated by rotary evaporation under vacuum. Each sample was concentrated to about 0.5 mL. Interfering compounds were removed by liquid-solid chromatography using 2:1 silica-alumina column. Two fractions were eluted. Fraction I (40 mL of hexane) contained n-alkanes, hopanes and steranes (Merck Corp., Germany), while fraction II (100 mL of DCM-hexane (1:1)) contained the priority PAHs. Then under a gentle stream of nitrogen, fraction I and fraction II were reduced almost to dryness and redissolved with 100 μL n-hexane. The two fractions were analyzed on GC-MS (Agilent Corp., 6890 − 5973 N, USA).

### BALF cell differentiation, cytokine quantification and carbon loading measurement

Rat’s lung was lavaged with 2 ml saline for three times to obtain a total volume of about 5 ml bronchoalveolar lavage fluid (BALF). BALF cells were collected then stained with hematoxylin and eosin (H&E) for differential counting of leukocytes under microscopy. At least 400 cells per slide were counted. Multiple cytokines in the BALF supernatant were measured by ELISA (R&D Systems, USA). Under light microscope, high-magnification figures were taken to measure the area of black carbon phagocyted by macrophages. The quantification analysis was performed with ImageScope software (Leica Biosystems, v.11, German). At least 10 fields in each slide were examined in a blinded manner.

### Histological evaluation

After obtaining the BAL fluid, right upper lungs were removed and fixed in 10% buffered formalin. The tissue samples were cut into 4-μm sections and affixed onto microscope slides and deparaffinized. Lung sections were stained with H&E for morphologic analysis. For identification of mucus-containing cells, Periodic Acid-Schiff (PAS) staining was applied. PAS-positive cells were counted in superficial epithelia of the small airways (perimeter < 1000 μm). Quantification analysis was determined by dividing the PAS-positive cells by the length of basement membrane (BM) of the small airways. For immunohistochemistry evaluation, sections were incubated with mouse monoclonal primary antibody, MUC5AC (Abcam, #Ab3649, UK), at 4 °C overnight. Following washes, the sections were measured by a two-step detection system (ZSGB-Bio, #PV-9000, China) according to manufacturer’s instructions. Subepithelial fibrosis was determined by evaluation of Masson’s Trichrome staining of the lung sections. Fibrotic area surrounding the small airways (perimeter < 1000 μm) was determined by image-pro plus 6.0 software (Media Cybernetics, USA). The total fibrotic area divided by the length of basement membrane (BM) was calculated. For evaluating airway remodeling, immunofluorescent staining was utilized. Sections were incubated with mouse monoclonal primary antibody, α-SMA (Novus Biologicals, #NB300-978, USA), at 4 °C overnight. Following washes, the sections were incubated with a secondary antibody (Abcam, #ab6881, UK). The sections were viewed under a laser confocal microscope (Nikon, C1Si, Japan).

### Morphometrical analysis of different airway compartments

The airway structure of rats differs significantly from that of humans. According to previous reports [26, 27], the small airway of rats was defined as perimeter of basement membrane (Pbm) ≤ 1000 μm, whereas the large-size airways were defined as Pbm of > 2000 μm, respectively (Supplementary Fig. 1). In this study, the small airways of rats included membranous bronchioles and respiratory bronchioles. Panoramic images of HE-stained lung section were acquired via Aperio digital pathologic image scanner (Leica Biosystems, German). The morphological analysis of the lung was performed using ImageScope viewing software (Leica Biosystems, German). According to a previous study in quantifying the area of airway wall [28], the total airway wall area (WAt) of each airway was calculated as WAt = Outer wall area (WAo) - Inner wall area (WAi). The relative area of each airway was calculated by dividing WAt by the length of the basement membrane (BM) of the airway. At least 10 different airways per section were analyzed. The mean linear intercept (MLI), a measurement of interalveolar septal wall distance, was determined using a reticule with a Thurlbeck grid comprising of 5 lines (each 550 μm long), with at least 10 fields assessed per section. The MLI was calculated through dividing the length of the line by the number of alveolar wall and gridline interception counted. At least 10 different peripheral lung parenchyma per section were analyzed.

### Determination of inflammatory cells in lung tissue

Macrophages and neutrophils were labelled with specific marker Ibal-1 (Wako, #019-19741, Japan) and Neutrophil elastase (Abcam, #ab310335, UK), respectively, using the standard immunohistochemistry (IHC) staining protocol. For eosinophil-specific staining, sections were stained for 30 min with chromotrope 2R (Sigma-Aldrich, #4197-07-3, USA) solution (1% chromotrope 2R in 5% phenol), by which the eosinophils were specifically stained in red. Another specific staining with 1% toluidine blue (Sigma-Aldrich, #6586-04-5, USA) was utilized to detect mast cells (MCs) cytoplasmic granules. All sections were examined under light microscopy. The numbers of Ibal-1 positive cells, elastase positive cells, eosinophils and mast cells were quantified according to the NIH Image Analysis system (National Institutes of Health, Bethesda, MD). The inflammatory cells around the airways were expressed as cells per millimeter (mm) basement membrane. At least 10 fields in each slide were examined in a blinded manner.

### Measurement of lung hydroxyproline content and systemic oxidative stress

The lower right lungs were analyzed for hydroxyproline content as an estimate of collagen content using Colorimetric Assay Kit (BioVision, #K555-100, USA). Detailed method has been described in our previous study [29].

Blood samples were taken from the left heart using a syringe with 25G needle and collected into tubes, then left to clot at 4ºC followed by centrifugation at 4ºC, 2400 g, for 10 min. Serum samples, stored at -80ºC before analysis, were analyzed for malondialdehyde (MDA) and 8-hydroxy-2’-deoxyguanosine (8-OHdG) using published HPLC and LC-MS/MS method [30].

### Lung function measurement

Lung function was measured using the Forced Pulmonary Maneuver System (Buxico Research Systems, USA). Rats anesthetized with pentobarbital (60 mg/kg body), were tracheostomized, intubated and placed in the body chamber of the system. The average breathing frequency was set to 80 breathes/min. Resistance and compliance were determined by measuring tracheal pressure and flow continuously during ventilation. To determine the total lung capacity (TLC), a quasi-static pressure volume maneuver was performed, by which the lungs were inflated to a standard pressure of + 30 cm H_2_O and then slowly exhales until a negative pressure of -30 cm H_2_O is reached.

### Statistical analysis

For the comparison of BALF cell count, pathological parameters, infiltration of inflammatory cells, level of inflammatory mediators, lung function parameters and carbon loading areas, data were shown as mean (95% CI). One-way analysis of variance (ANOVA) test and Tukey’s multiple comparisons test were used to determine the differences among groups. Statistical differences between the two groups were analyzed by Student t-test. Statistical significance was set at *P* < 0.05. All analyses were performed with the Prism software (GraphPad Software, version 9.0, USA).

## Results

### Exposure characteristics

**Fig. 2:**
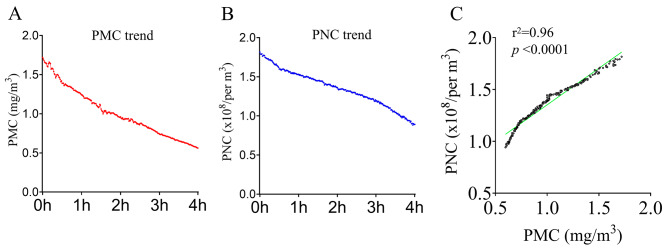
Changing trend of DEP concentrations during exposure period. The changing trends of particle mass concentration (PMC, **A**) and particle number concentration (PNC, **B**) during 4 h DEP exposures were shown. Correlation analysis showed that the changing trend of PMC was significantly correlated with that of PNC during the exposure period (**C**, r^2^ = 0.96, *p* < 0.0001)

During the DEP exposure period, changing trends of particle mass concentration (PMC) and particle number concentration (PNC) were shown in Fig. 2. A total of 240 data points were measured during 4-hour DEP exposure. The average levels (95% CI) of PMC and PNC were 1.03 mg/m^3^ (range: 0.99–1.07 mg/m^3^) and 1.36 × 10^8^ (1.33 ~ 1.38 × 10^8^ /m³), respectively. In addition, PMC was correlated significantly with PNC during the exposure period (*p* < 0.0001). The PMC in the exposure chamber was nearly 25-fold compared to that in filtered air environment of 0.042 mg/m^3^ (0.036–0.047 mg/m^3^).

The chemical composition analysis showed that OC and EC accounted for 76.74% and 20.69% of the total DEP mass, respectively. We identified 25 metal elements and 25 PAHs, accounting for 0.71% and 0.37% of DEP mass, respectively. The most abundant metal elements were Na and Ca, followed by Mg, Al and K. The presence of redox-active transition metals (e.g., Fe, Cu, Zn, Ag, Cr, Mn, Cd) was evident (Table 1). Among the PAHs, benzo(ghi)perylene, Chrysene/Tri-phenylene and benzo(a)pyrene were most abundant (Table 2).

**Table 1:** Concentrations of metal elements in DEP

Element	Li	Be	Na	Mg	Al	K	Ca	V	Cr	Mn	Fe	Co	Cu
Con. ng/m^3^	0.4	0.01	4718.1	908.9	584.8	202.0	1829.7	0.06	6.6	1.80	35.7	0.05	0.70
Element	Zn	Ga	As	Se	Rb	Sr	Ag	Cd	Cs	Ba	Tl	Pb	Total
Con. ng/m^3^	1.2	0.1	0.1	0.1	0.3	1.1	0.1	0.1	0.03	3.0	0.001	0.1	8295.1

**Table 2:** Concentrations of PAHs in DEP

PAH	Acenaphthyl-ene	Acenaphthe-ne	Fluorene	Phenanthrene	Anthracene	Fluoranthene	Acephenant-hrylene	Pyrene	Retene
Con. ng/m^3^	1.76	2.07	1.12	128.25	6.25	191.08	21.45	271.71	3.70
PAH	Benzo(ghi)-fluoranthene	Cyclopenta-(cd)pyrene	Benz(a)anth-racene	Chrysene/Tri-phenylene	Benzo(b)flu-oranthene	Benzo(k)fluo-ranthene	Benzo(j)flu-oranthene	Benzo(e)p-yrene	Benzo(a)py-rene
Con. ng/m^3^	156.74	9.37	168.98	435.13	380.93	384.32	23.00	417.28	418.19
PAH	Perylene	Indeno(cd)- fluoranthene	Indeno(cd)- pyrene	Dibenzo[a,h]-anthrancene	Picene	Benzo(ghi)- perylene	Coronene	Total	
Con. ng/m^3^	0.80	163.83	388.62	42.08	22.57	510.87	206.10	4356.19	

### DEP exposure induced COPD-like pathologies

To determine whether DEP exposures can induce COPD-like pathologies, we performed histological evaluations. We did not observe significant changes of the total wall area (WAt) in large airways following DEP exposures (Fig. 1. **C-D**). In contrast, there were about 2-fold increase in WAt of the small airways following DEP exposures for 2 weeks [WAt/BM, 35.2 (32.5 ~ 37.9) μm^2^ vs. 20.2 (16.7 ~ 23.8) μm^2^], 4 weeks [39.2 (34.3–40.1) μm^2^ vs. 21.5 (19.5 ~ 23.6) μm^2^], and 8 weeks [41.8 (35.8–47.9) μm^2^ vs. 19.2 (18.0-20.4) μm^2^] (Fig. 1. **E, F**). Following 2-week DEP exposure, MLI in the alveoli region showed an increase trend without statistical significance, as compared to the filtered air control. Following the longer exposure durations, however, MLI had increased significantly [89.7 (81.1–98.4) μm vs. 48.1 (45.1–51.3) μm, for 4-wk exposure] and [101.8 (87.3–116.0) μm vs. 51.1 (45.5 ~ 56.7) μm, for 8-wk exposure] (Fig. 1. **G, H**).

### Carbon loading is elevated in alveolar macrophages

**Fig. 3:**
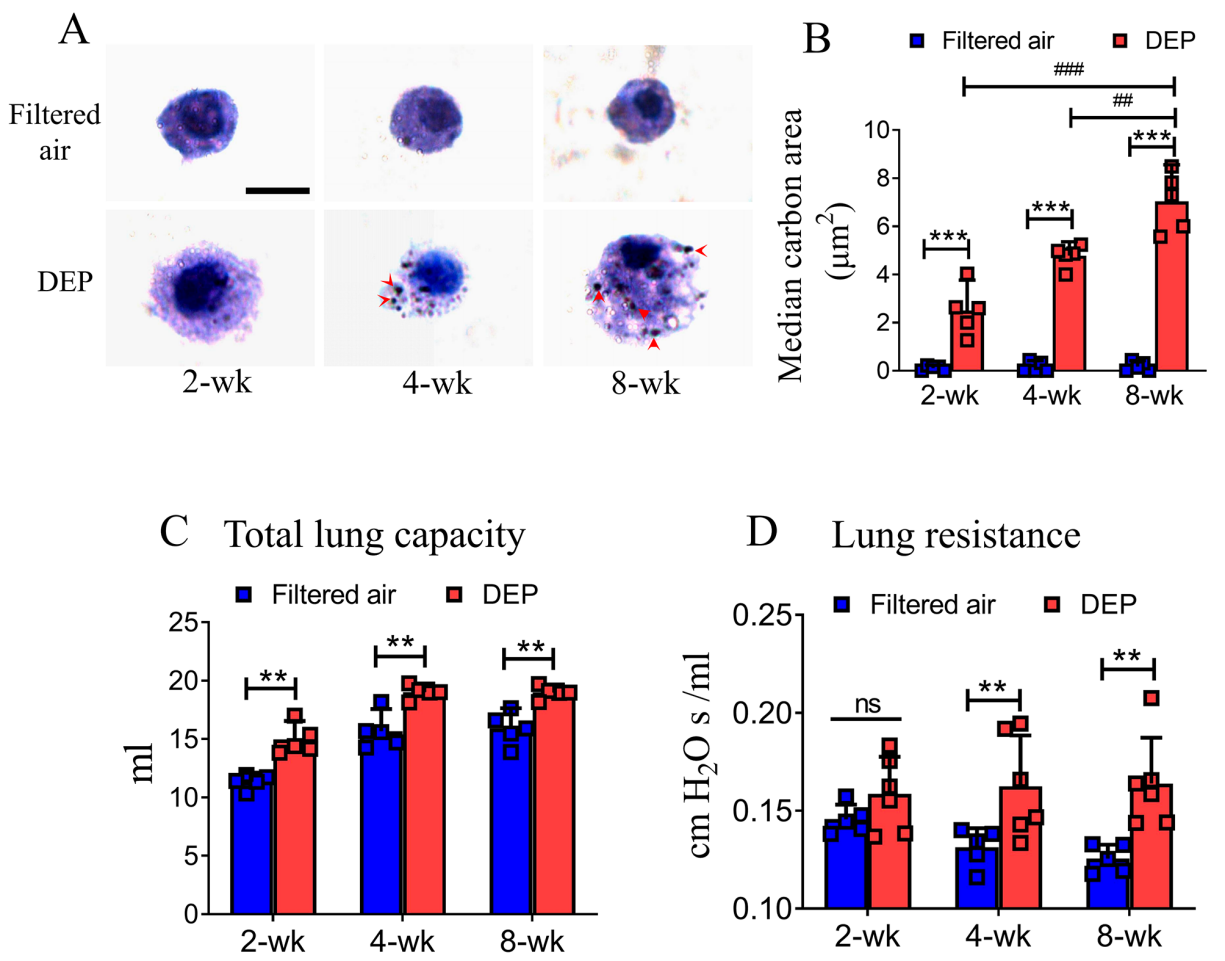
Carbon loading in alveolar macrophages and lung function parameters. Carbon uptake areas in BALF macrophages (**A**, red arrows) following DEP exposures for 2-wk, 4-wk and 8-wk significantly increased, in a dose-dependent manner, compared to carbon uptake areas following filtered air exposure (*** *p* < 0.001, ## *p* < 0.01, ### *p* < 0.001, **B).** Bar = 8 μm. Total lung capacity (TLC) increased significantly following 2-wk, 4-wk and 8-wk DEP exposures as compared to filtered air controls, respectively (** *p* < 0.01, **C**). Also, lung resistance increased significantly following 4-wk and 8-wk DEP exposures, respectively (** *p* < 0.01, **D**). Data were expressed as mean (95% CI). ns = not significant

In order to quantify the exposure level upon inhalation exposure to DEP, we measured the area of carbon loading in rats’ alveolar macrophages. The results showed that inhaled elemental carbon (EC or black carbon) was taken up by alveolar macrophages in a dose-dependent manner. The areas showing black carbon uptake in BAL macrophages were increased significantly to 2.5 (1.2 ~ 3.8) μm^2^, 4.8 (4.2 ~ 5.4) μm^2^, and 7.0 (5.5 ~ 8.5) μm^2^ following DEP exposures for 2, 4, and 8 weeks, respectively, compared to those exposed to filtered air (Fig. 3, A-B).

### DEP exposure induced lung function impairments

To determine the changes in lung function, we measured lung function parameters of rats following exposures to DEP or filtered air. Total lung capacity (TLC) was significantly increased following each of the 3 DEP exposures in reference to the filtered air control: [14.9 (13.4–16.5) ml vs. 11.3 (10.2–12.4) ml for 2-wk exposure], [19.0 (18.3–19.7) ml vs. 15.7 (13.8–17.6) ml for 4-wk exposure], and [19.0 (18.0–20.0) ml vs. 15.9 (14.2–17.6) ml for 8-wk exposure] (Fig. 3. **C**). Also noted were the increases in lung resistance (Rl) following the 4-wk or the 8-wk DEP exposure (Fig. 3. **D**). Although 2-wk DEP exposure appeared to increase Rl without a statistical significance, longer-duration exposures resulted in significant Rl increases [0.16 (0.13–0.18) vs. 0.13 (0.12–0.14) cm H_2_O s/ml for 4-wk exposure] and [0.16 (0.14–0.19) vs. 0.13 (0.11–0.13) cm H_2_O s/ml for 8-wk exposure].

### DEP exposure induced remodeling and fibrosis of the peripheral lung

**Fig. 4:**
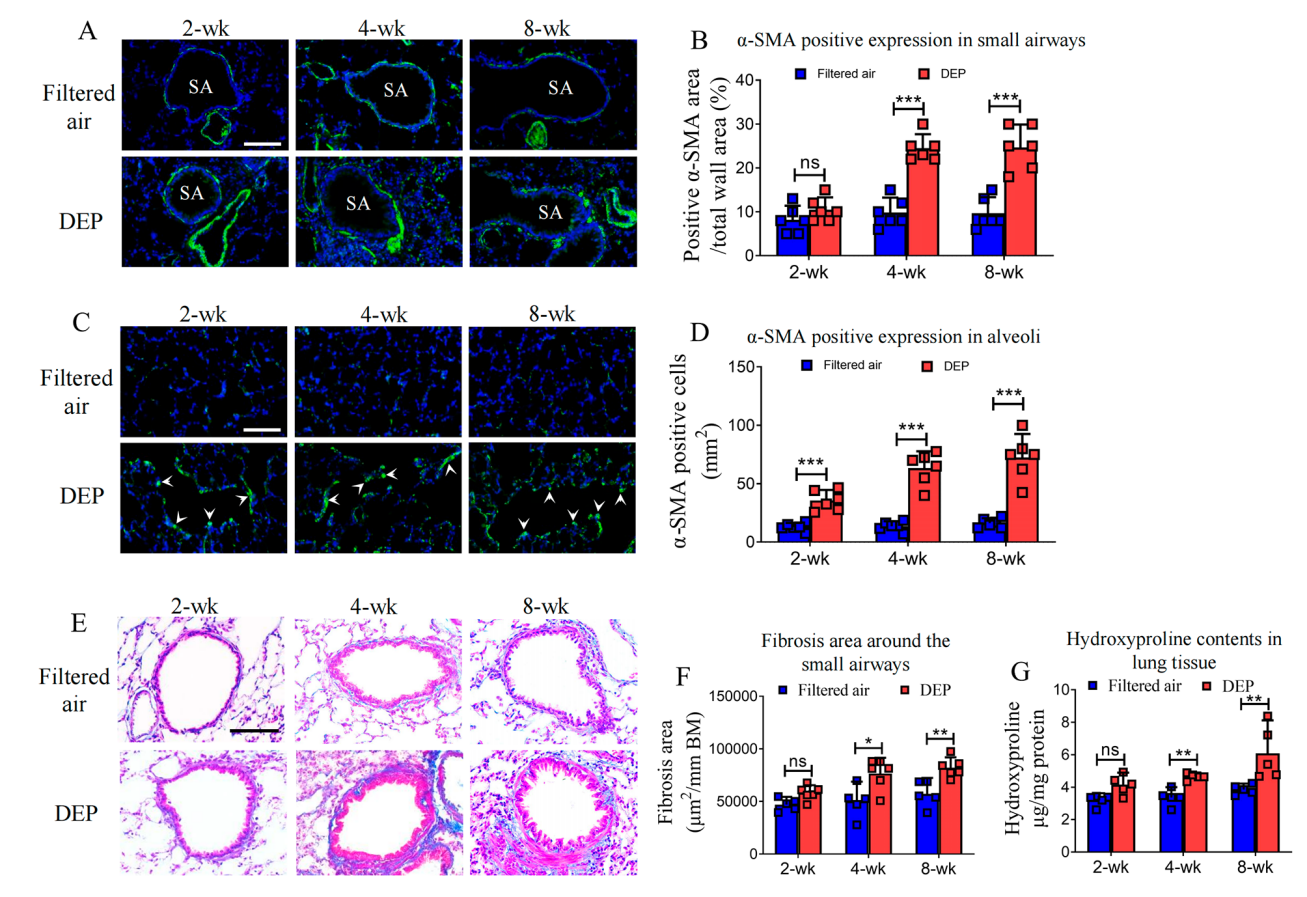
Remodeling and fibrosis of the peripheral lung. Of the small airways, the relative expression of α-SMA (stained in green) of smooth muscle to total wall area increased significantly in DEP exposure for 4-wk and 8-wk, respectively (*** *p* < 0.001, **A-B**). In the alveolar region, the number of α-SMA positive cells (white arrow head, **C**) increased significantly post-DEP exposures (*** *p* < 0.001, **D**). The fibrosis areas (stained in blue, **E**) around the small airways also significantly increased following 4-wk and 8-wk DEP exposures, respectively (* *p* < 0.05, ** *p* < 0.01, **F**). The contents of hydroxyproline in lung tissue significantly increased in rats exposed to DEP for 4 weeks and 8 weeks, respectively (** *p* < 0.01, **G**). Data were expressed as mean (95% CI). Bars in the immunofluorescent staining images = 50 μm, while bar in the Masson Trichrome staining image = 100 μm. SA: small airways, ns = not significant

To determine the remodeling of peripheral lung, we measured α-SMA expression using rats’ lung slice by immunofluorescent staining. Of the small airways, positive expressions of α-SMA (positive area/total airway wall area) increased significantly following DEP exposures for 4 weeks [24.5% (21.3-27.6%) vs. 9.8% (6.4-13.2%)] and 8 weeks [24.7% (19.5-30.0%) vs. 9.7% (6.0-13.3%)], respectively (Fig. 4, A-B). In the alveolar region, relative to the filtered air control conditions, the number of positive cells expressing α‐SMA increased significantly following DEP exposures over 2 weeks [35.7 (26.7–44.6) cells/mm^2^ vs. 13.0 (9.5–16.4) cells/mm^2^], 4 weeks [63.3 (48.9–77.8) cells/mm^2^ vs. 13.7 (9.4–17.9) cells/mm^2^], and 8 weeks [72.5 (52.4–92.5) cells/mm^2^ vs. 16.3 (12.1–20.4) cells/mm^2^], respectively (Fig. 4, C-D).

To determine lung fibrosis upon DEP exposure, we measured lung hydroxyproline content and subepithelial fibrosis of different airway compartments. Following the 2-wk DEP exposure, areas of fibrosis around the small airways tended to increase in reference to the filtered air control, as we did not observe a difference of statistical significance. With longer DEP exposure durations, however, we observed significant increases of fibrotic area around the small airways for 4-week [7.7 × 10^4^ (6.2 × 10^4^ ~ 9.2 × 10^4^) μm^2^ vs. 5.0 × 10^4^ (3.1 × 10^4^ ~ 6.9 × 10^4^) μm^2^] and for 8-week [8.2 × 10^4^ (7.2 × 10^4^ ~ 9.2 × 10^4^) μm^2^ vs. 5.7 × 10^4^ (4.2 × 10^4^ ~ 7.2 × 10^4^) μm^2^] (Fig. 4. **E-F**). Consistently, levels of hydroxyproline protein increased significantly following DEP exposures for 4 weeks [4.7 (4.4–4.9) μg/ mg vs. 3.4 (2.7-4.0) μg/mg] and 8 weeks [6.1 (4.0-8.1) μg/mg vs. 3.9 (3.5–4.2) μg/mg] (Fig. 4. **G**). In sharp contrast, DEP exposures of any duration did not result in significant fibrotic changes of the large airways (Supplementary Fig. 2).

### DEP exposure induced mucus hypersecretion and goblet cell hyperplasia of the small airways

**Fig. 5:**
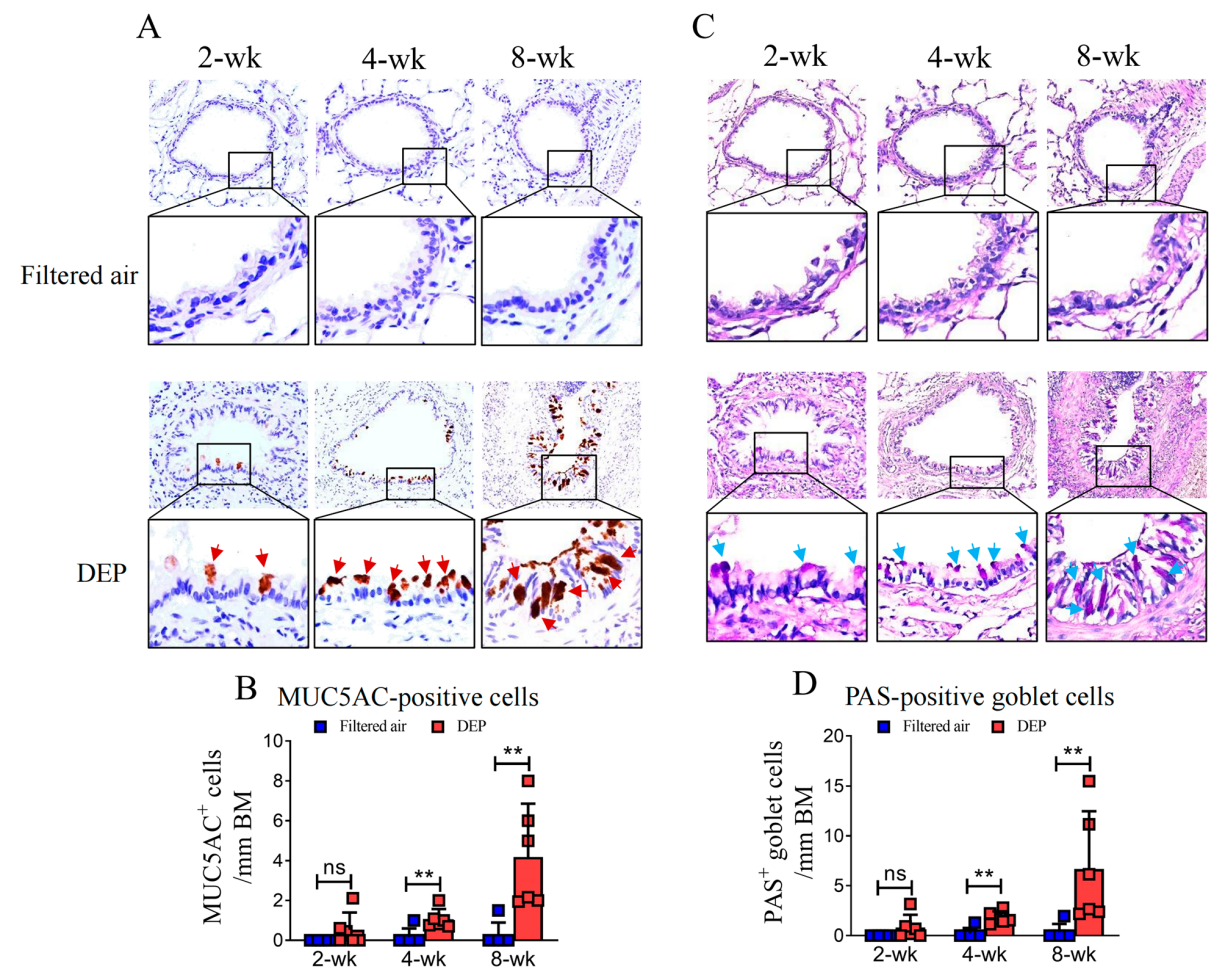
Mucus production and epithelial cell morphology of the small airways. The MUC5AC-immunopositive cells (red arrows, **A**) and PAS-positive goblet cells (blue arrows, **C**) were indicated. Quantification analysis showed that the number of MUC5AC positive cells of the superficial epithelia was increased significantly following DEP exposures over 4-wk and 8-wk (** *p* < 0.01, **B**). The number of PAS positive cells was also increased significantly following DEP exposures for 4-wk and 8-wk (** *p* < 0.01, **D**). Data were expressed as mean (95% CI). Magnification of the lower panel images in A and C, 1000 x, ns = not significant

To evaluate the epithelial cell morphology and mucus production, we performed Periodic Acid-Schiff (PAS) staining and immunohistochemical staining with anti-MUC5AC antibody using rats’ lung slice. As shown in Fig. 5A-B, the number of MUC5AC-positive cells in the small airway epithelia increased significantly following exposure to DEP for 4 weeks [1.06 (0.55–1.57) cells/ mm BM vs. 0.17 (0-0.60) cells/ mm BM] and 8 weeks [4.18 (1.51–6.85) cells/ mm BM vs. 0.25 (0-0.89) cells/ mm BM], indicating the involvement of mucus hypersecretion in this model. Consistently, relative to the filtered air controls, 4-week and 8-week DEP exposures caused a significant increase in the number of PAS-positive goblet cells of the superficial epithelia [4-week: 1.81 (1.18–2.44) cells/ mm BM vs. 0.22 (0-0.78) cells/ mm BM; 8-week: 6.67 (0.87–12.47) cells/ mm BM vs. 0.33 (0-1.17) cells/ mm BM], respectively (Fig. 5C, D), indicating the presence of airway goblet cell hyperplasia.

### DEP exposure induced significant inflammatory cell recruitment to the lung

**Fig. 6:**
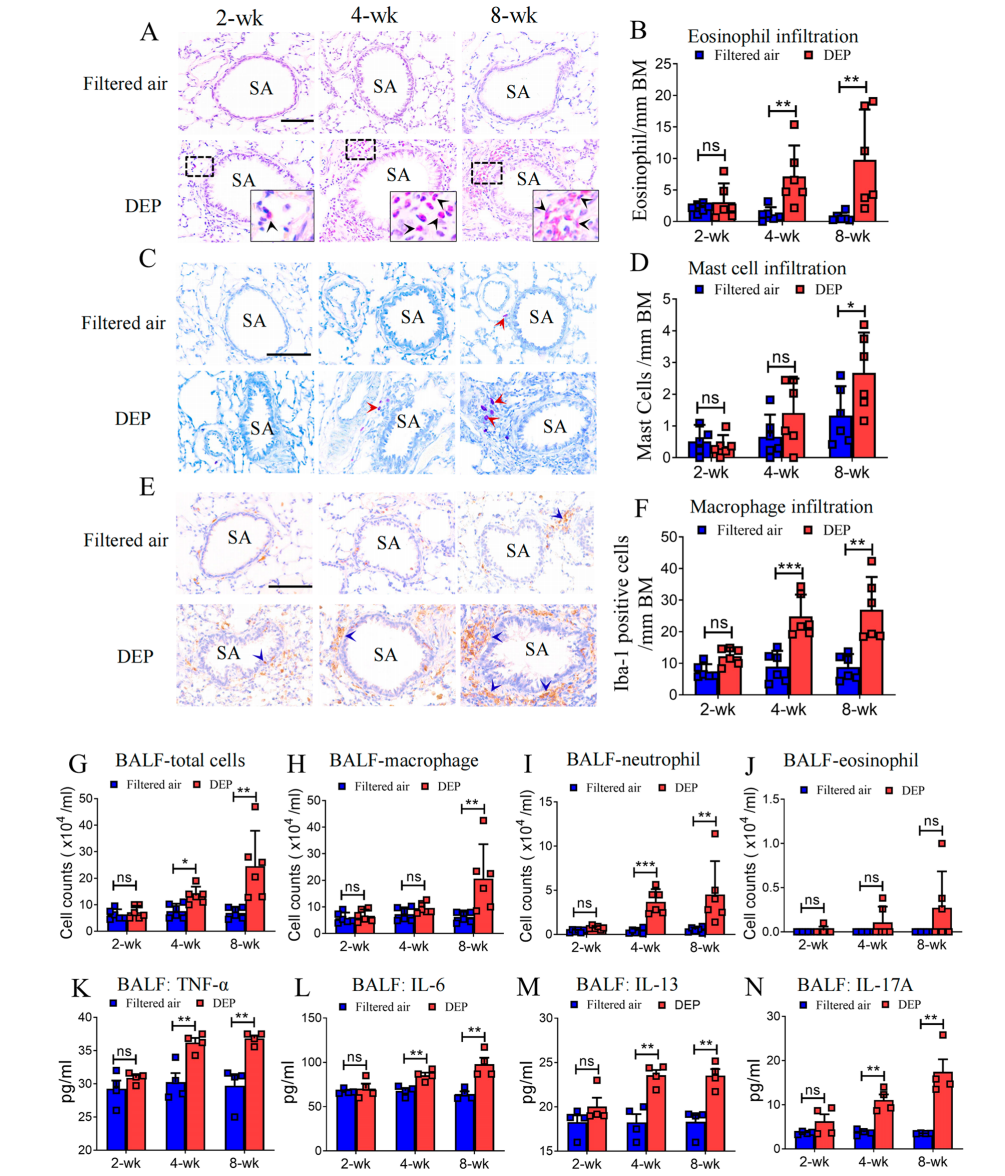
Recruitment of inflammatory cells to the small airways and inflammatory mediators in BAL fluid. Eosinophil (black arrow head, **A**) infiltrated around small airways increased significantly in rats exposed DEP for 4 weeks and 8 weeks (** *p* < 0.01, **B).** For the mast cells (red arrows head, **C**), exposure of rats to DEP for 8 weeks (* *p* < 0.05, **D**), but not for shorter durations, demonstrated a significant increase around the small airways. The infiltrations of Iba-1 immuno-positive macrophages (blue arrow head, **E**) were significantly increased in small airways following 4-wk (*** *p* < 0.001) or 8-wk DEP exposure (** *p* < 0.01), as compared to the filtered air controls, respectively (**F**). Bar in the images (Panel A, C, E) = 100 μm. ns = not significant. SA = small airways. Cell differentials in BALF showed significant increases of total leukocyte, macrophage as well as neutrophil, but not eosinophils, following 8-week DEP exposure, as compared to filtered air control, respectively (** *p* < 0.01, ns, not significant, **G-J**). DEP exposure for 4-wk or 8-wk significantly increased BALF concentrations of TNF-α, IL-6, IL-13 and IL-17 A, respectively (** *p* < 0.01, ns, not significant, **K-N**). Data were expressed as mean (95% CI)

To determine the infiltrations of various immune cells, we performed specific staining or immunohistochemistry staining in lung slice. Exposure to DEP induced potent inflammatory responses of the peripheral lung. A dramatical increase of eosinophils around the small airways in DEP-exposed rats was observed. As compared to the filtered air controls, 4-wk and 8-wk DEP exposures caused a 6.5-fold increase [7.2 (2.2–12.6) vs. 1.1 (0.4–2.6)/mm BM] and a 14.0-fold increase [9.9 (1.8–17.8) vs. 0.7 (0.0-1.37)/mm BM] in eosinophils infiltration around the small airways, respectively (Fig. 6. **A-B**). In addition, recruitment of eosinophils to the alveolar region and pulmonary arteries was also evident. (Supplementary Fig. 3)

As another immune effector cell, we observed significant infiltrations of mast cells in the submucosa of small airways in rats exposed to DEP for 8-week [2.7 (1.4–3.9)/ mm BM vs. 1.3 (0.4–2.3)/ mm BM] as compared to filtered air control (Fig. 6C-D). We also found significant increases of Ibal-1 positive macrophages in submucosal layer of the small airways following exposures to DEP for 4-week [24.9 (19.3–30.5)/mm BM vs. 8.9 (4.5–12.7)/mm BM] and 8-week (27.3 (16.8–37.3)/mm BM vs. 8.8 (5.0-12.9)/mm BM) (Fig. 6E-F). However, we did not find evident infiltration of neutrophil in rats’ peripheral lung. As compared to filter air controls, exposure of rats to DEP over 2-week to 8-week did not induce significant increases in neutrophil infiltration around the small airways (Supplementary Fig. 4).

### Increased level of inflammatory mediators in BALF upon DEP exposures

To determine the inflammatory response of the rats’ lung, we measured the inflammatory cell differential and cytokine levels in BALF. Compared with those following the filtered air exposures, counts of total cells, macrophages, and neutrophils in BALF were all significantly higher following the 8-wk DEP exposure (*p* < 0.01) (Fig. 6, G-I); neutrophil counts were also significantly increased following the 4-wk DEP exposure (*p* < 0.001). We observed an increasing trend of eosinophil count in BAL fluid following DEP exposures, though the difference did not reach statistical significance (*p* > 0.05) (Fig. 6, J). Compared to the filtered air controls, both 4-wk and 8-wk DEP exposures resulted in significant increases in BALF concentrations of pro-inflammatory cytokines, including TNF-α, IL-6, IL-13 and IL-17 A (Fig. 6, K-N). However, the concentration of IL-5 in BALF did not change significantly following DEP exposures (Supplementary Fig. 5).

### DEP exposure induced systemic oxidative stress

**Fig. 7:**
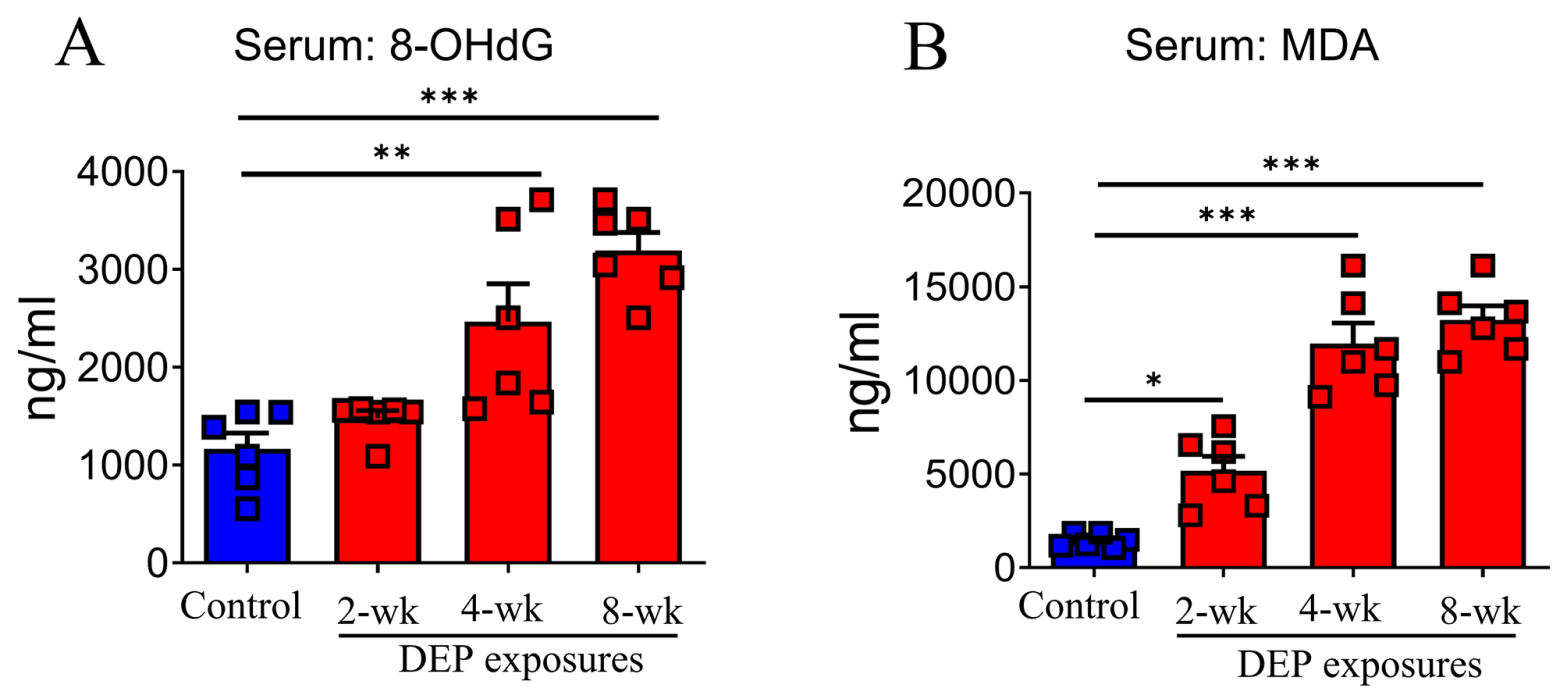
Oxidative stressor concentrations. As compared to filtered air control, the serum levels of 8-OHdG increased significantly in rats exposed to DEP for 4-wk or 8-wk (** *p* < 0.01, *** *p* < 0.001, **A**). Exposure of rats to DEP for 3 durations resulted in significant increases of serum MDA levels, as compared to the control, respectively (* *p* < 0.05, *** *p* < 0.001, **B**). Data were expressed as mean (95% CI)

Systemic effects of DEP exposures were assessed using serum malondialdehyde (MDA, a product of lipid peroxidation) and 8-hydroxy-deoxyguanosine (8-OhdG, a product of DNA oxidation). Compared to the filtered air control, 4-wk and 8-wk DEP exposures induced a significant increase in 8-OhdG concentration, respectively (Fig. 7. **A**). We also observed a significant increase in serum MDA concentration following DEP exposures of any duration (Fig. 7. **B**).

## Discussion

The principal novel finding of the present study is that repeated DEP exposures caused small airway remodeling as the initial site of injury, consequently leading to the development of COPD with an eosinophilic phenotype in a rat model. Our rat model (healthy SD rats) represents the healthy lung. The DEP, containing common elements and organic compounds, was generated freshly from a diesel engine, represented a surrogate of traffic related urban PM_2.5_. The present study, on the other hand, was designed to mainly elucidate the pathophysiologic processes underlying the development of COPD upon DEP exposure.

Emerging evidence in humans has suggested that chronic PM_2.5_ exposure may affect small airway structure and function. For example, Churg et al. found an association between high level of ambient particulate matter and small airway remodeling in residents living near traffic roads [31]. Wang et al. showed that long-term exposure to ambient PM_2.5_ or black carbon correlated significantly with computed tomographic defining emphysema in adults [32]. Liu et al. suggested that chronic exposure to diesel exhaust may be related to small airway wall thickening in diesel engine testers [33]. In the present study, we confirmed the causal effects of DEP exposure on small airway remodeling as well as lung emphysema and associated lung function impairments.

Our morphologic analysis showed that relative total wall area (WAt) of the small airways in DEP-exposed rats was about 2-fold larger as compared to those in the filtered air control. Also noted were the increased expression of α-SMA in small airway wall. These interesting pathologic phenomena indicate that small airway remodeling remained an earlier adverse event following DEP exposure. In addition, fibrosis of the lung was evident in DEP-exposed rats, as indicated by the increased level of hydroxyproline in lung homogenates. Furthermore, we found significant increase in fibrotic area of the small airways but not larger airways, suggesting small airways are more vulnerable to the adverse effects induced by DEP. Exposure of rats to DEP induced phenotypic changes of goblet cell hyperplasia and mucus hypersecretions in small airways. Being the place where the gases shift from bulk flow to diffusion occurs, the small airway has been hypothesized as the primary target for the impaction of PM_2.5_ inhaled into the lungs [34, 35]. One the other hand, it has been shown that the mucus and ciliated cells decreased from the large conducting airways to peripheral airways [36]. In this case, the small airways might be less potent in the mucociliary clearance of inhaled DEP. Indeed, inhalation of aerosol particles ranged in smaller sizes (typical of DEP) has been shown to deposit mainly in the small airways of rodents [21, 22]. Taken together, small airway impairment might represent an important pathophysiologic process in DEP-induced COPD.

Exposure of rats to DEP also induced emphysematous alterations of the lung, as revealed by the significant enlargement of the alveoli. Although the presence of lung emphysema following 4-week DEP exposure was later than that of small airway remodeling following 2-week DEP exposure, increased expressions of α-SMA was also observed in the alveolar region. As a phenotypic marker of epithelial to mesenchymal transition (EMT), alterations of α-SMA expression have been implicated in peripheral lung of COPD patients [37, 38] or in cigarette-induced COPD mice [39]. Thus, the phenotypic changes in alveolar region might be another pathological process involved in DEP-induced COPD.

DEPs are mostly ultrafine or nanoscale particles. In this study, we showed that black carbon was phagocyted by alveolar macrophages in a dose-dependent fashion (the longer DEP exposure was, the larger the carbon-loaded area was.) This indicates that DEP reached deep into the peripheral airways. Due to their small particle size, DEP posed large relative surface area that allowed for containing various toxic substances. Through chemical analysis, we identified various metal elements and PAHs that accounted for 0.71% and 0.37% of the total mass of DEP, respectively. Transitional metals, especially Fe, are potent oxidative stressors in inducing the generation of reactive oxygen species (ROS) [40]. DEP containing PAHs or oxygenated-PAHs, such as Benzo(a)pyrene and quinone, have been shown to stimulate the releases of ROS in airway epithelial cells as well as macrophages [41, 42]. Excessive ROS produced in target cells can heighten the peroxidation of cell membrane lipids and attacks DNA molecules, which lead to the elevated production of MDA and 8-OHdDG that seen in this model.

Inflammatory responses of the airways are the core feature of chronic airway diseases. In the present study, we found significant airway inflammation characterized by increased numbers of macrophages and neutrophils in BALF following DEP exposure, consistent to previous studies [43, 44]. In addition, a dramatic infiltration of eosinophils to the peripheral lung, including the small airways and alveoli, was observed in DEP-exposed rats. Accumulation of eosinophils in lung tissues has been shown to contribute to airspace enlargement and alveolar destruction in mice [45]. Significantly increased levels of type 2 cytokines, e.g., IL-13, IL-4 and TNF-α, but not IL-5, and an increasing trend of eosinophil count were exhibited in the BALF. Eosinophils are terminally differentiated, bone marrow-derived, granule-containing leukocytes, which are potent drivers of inflammatory damage, airway remodeling and fibrosis through secreting type 2 cytokines as well as various enzyme-containing granules [46]. Although the exact mechanisms underlying DEP-induced lung eosinophilia are unclear, we inferred that DEP-induced eosinophil recruitment to the peripheral lung might be related to an IL-5 independent mechanism. For example, previous studies have shown that type 2-promoting cytokines, such as IL-33 [47] and TSLP [48] released by DEP-exposed airway epithelial cells, are involved in pollutant-induced eosinophilic airway inflammation. In addition, DEP per se may act as allergen carriers that absorbed various allergens, such as pollen, dust mites and pathogens [49, 50], which might enhance eosinophil recruitment to the peripheral lungs in DEP-exposed rats. Likewise, previous studies have shown that eosinophilic inflammation was evident in biomass smoke-related COPD, but the majority of cell population in the sputum was neutrophil [17, 51], which is consistent with our current findings. In addition, the lack of neutrophil recruitment to the airway mucosa might be due to the rapid transition of this cell type from tissue to the airway lumen that has been observed in COPD patients [52].

COPD is a complex and heterogeneous disease that may result from different etiologies and risk factors. Two main causative agents, such as cigarette smoke (CS) and biomass smoke (BS), have been widely applied to model COPD in experimental studies. Pathologic findings showed that emphysema was the major phenotype in CS-related COPD, while small airway remodeling and fibrosis were predominant in BS-related COPD [53, 54]. As another risk factor for incident COPD, we showed that DEP-related COPD could manifest as small airway remodeling and emphysema, along with significant eosinophil infiltrations. Although BALF neutrophils were the primary cell type found to be increased following DEP exposure, we did not observe evident neutrophilic infiltration in the peripheral lung. We propose that eosinophil recruitment to the small airways and alveolar regions might represent a distinct feature in DEP-related COPD.

We conducted the experiments in a diesel chamber with steady meteorological conditions. There were no notable infections or deaths of rats during DEP exposures. Being the first in vivo model to demonstrate the pathophysiologic features of DEP-induced COPD, however, the present study has some limitations. First, although injuries of the small airways were evident following DEP exposures, the molecular mechanisms underlying the involved pathophysiologic processes remain to be further elucidated. Also, the pathologic alterations and inflammatory phenotype in long-term DEP exposure need future investigations. Second, freshly generated DEP contains a mixture of various constituents, some of which have been identified in this study. However, the oxidative potential and toxicity between fresh DEP and aged DEP in atmospheric environment are different [55]. In this case, caution should be made when explaining the adverse effects of fresh DEP and real-world PM_2.5_. Third, in addition to T2-driven inflammation, increased number of CD8 + T-lymphocytes and Th1-driven inflammatory response has been shown to play important roles in the pathogenesis of COPD [56, 57]. We wish to evaluate these phenotypic changes in DEP-related COPD in the future. Finally, we did not take age into account in evaluating the adverse effects of DEP. Previous publications have shown that age can impact the response to particulate matter pollution exposure in a murine model [58, 59]. Hence, future studies are recommended to examine how age modifies the contribution of PM_2.5_ exposure to the development of COPD.

## Conclusions

In summary, this study adds novel findings to the existing knowledge on the role of particulate pollution and the role of the small airways in the pathophysiology and environmental etiology of COPD. Our findings suggest that DEP exposure contributes to the etiology of COPD with a distinct eosinophilic infiltration in the peripheral lung.

### Electronic supplementary material

Below is the link to the electronic supplementary material.

**Supplementary Material 1: Figure S1.** Whole lung morphologic showing large airway with perimeter > 2000 μm (LA, Red box), small airway with perimeter ≤ 1000 μm (SA, Blue box), pulmonary artery with diameter between 50–150 μm (PA, Blue box) and alveolar region (Green box). Bar = 1000 μm. **Figure S2.** Fibrosis quantification of the large airways. Fibrosis areas around the large airways with perimeter > 2000 μm showed an increasing trend, but did not reach statistical significance following 2wk to 8-wk DEP exposures, respectively. Bar in the Masson Trichrome staining image = 100 μm. n = 6 rats/group. ns = not significant. **Figure S3.** Recruitment of eosinophils (black arrows) to the alveolar region (Panel A) and pulmonary arteries (Panel C). Eosinophil infiltrated in the alveolar increased significantly in rats exposed DEP for 4 weeks and 8 weeks (B). Rats exposed to DEP for 4 weeks and 8 weeks, but not for shorter durations, demonstrated a significant increase of eosinophils around the PA (D). Bar in the C2R staining image = 100μm. * *p* < 0.05, *** *p* < 0.001. n = 6 rats/group. PA = pulmonary arteries. **Figure S4.** Representative images of IHC staining of neutrophils in small airways, showing the recruitment of neutrophils (red arrows) around the small airways in DEP exposure group and the control group, by exposure duration (Panel A). T-tests showed that neutrophil infiltration of the small airways did not reach statistical difference between the DEP exposure groups and filtered air controls (Panel B). Magnification of IHC staining image, lower panel, 1000x, n = 6 rats/group. ns = not significant. **Figure S5.** DEP exposures over the courses of 2-wk to 8-wk did not significantly change the concentrations of IL-5 in BALF. ns = not significant

## Data Availability

The raw data required to reproduce these findings are available within the article and the supplementary files. Data are available from the corresponding author upon reasonable request.

## List of abbreviations

α-SMA: Alpha smooth muscle actin
8-OHdG: 8-hydroxy-2’-deoxyguanosine
BALF: Bronchoalveolar lavage fluid
BM: Basement membrane
COPD: Chronic obstructive pulmonary disease
DEP: Diesel exhaust particle
EC: Elemental carbon
MDA: Malondialdehyde
MLI: Mean linear intercept
OC: Organic carbon
PAH: Polycyclic aromatic hydrocarbons
PM_2.5_: Particulate matter with aerodynamic diameter < 2.5 μm
PMC: Particle mass concentration
PNC: Particle number concentration
WAt: Total wall areas
