# Supplementary material for: Fine particulate matter contributes to COPD-like pathophysiology: experimental evidence from rats exposed to diesel exhaust particles
Source: Respir Res. 2024 Jan 4;25:14. doi: 10.1186/s12931-023-02623-y (PMC10765838; doi:10.1186/s12931-023-02623-y)
Supplement: Supplementary file 1 — Supplementary Material 1: Figure S1. Whole lung morphologic showing large airway with perimeter > 2000 μm (LA, Red box), small airway with perimeter ≤ 1000 μm (SA, Blue box), pulmonary artery with diameter between 50–150 μm (PA, Blue box) and alveolar region (Green box). Bar = 1000 μm. Figure S2. Fibrosis quantification of the large airways. Fibrosis areas around the large airways with perimeter > 2000 μm showed an increasing trend, but did not reach statistical significance following 2wk to 8-wk DEP exposures, respectively. Bar in the Masson Trichrome staining image = 100 μm. n = 6 rats/group. ns = not significant. Figure S3. Recruitment of eosinophils (black arrows) to the alveolar region (Panel A) and pulmonary arteries (Panel C). Eosinophil infiltrated in the alveolar increased significantly in rats exposed DEP for 4 weeks and 8 weeks (B). Rats exposed to DEP for 4 weeks and 8 weeks, but not for shorter durations, demonstrated a significant increase of eosinophils around the PA (D). Bar in the C2R staining image = 100μm. * p < 0.05, *** p < 0.001. n = 6 rats/group. PA = pulmonary arteries. Figure S4. Representative images of IHC staining of neutrophils in small airways, showing the recruitment of neutrophils (red arrows) around the small airways in DEP exposure group and the control group, by exposure duration (Panel A). T-tests showed that neutrophil infiltration of the small airways did not reach statistical difference between the DEP exposure groups and filtered air controls (Panel B). Magnification of IHC staining image, lower panel, 1000x, n = 6 rats/group. ns = not significant. Figure S5. DEP exposures over the courses of 2-wk to 8-wk did not significantly change the concentrations of IL-5 in BALF. ns = not significant [file 12931_2023_2623_MOESM1_ESM.pdf]

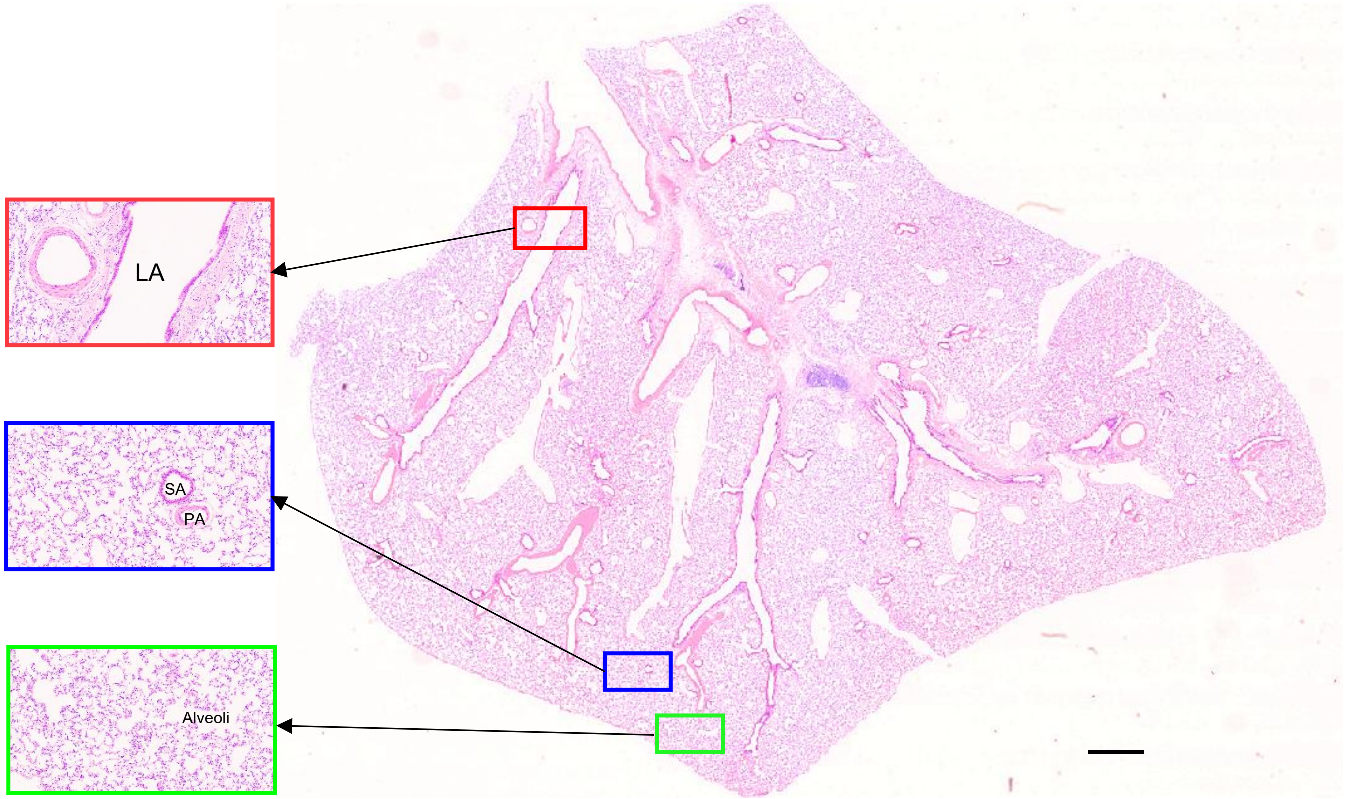

Figure S1. Whole lung morphologic showing large airway with perimeter > 2000 µm (LA, Red box), small airway with perimeter ≤ 1000 µm (SA, Blue box), pulmonary artery with diameter between 50-150 µm (PA, Blue box) and alveolar region (Green box). Bar=1000 µm.

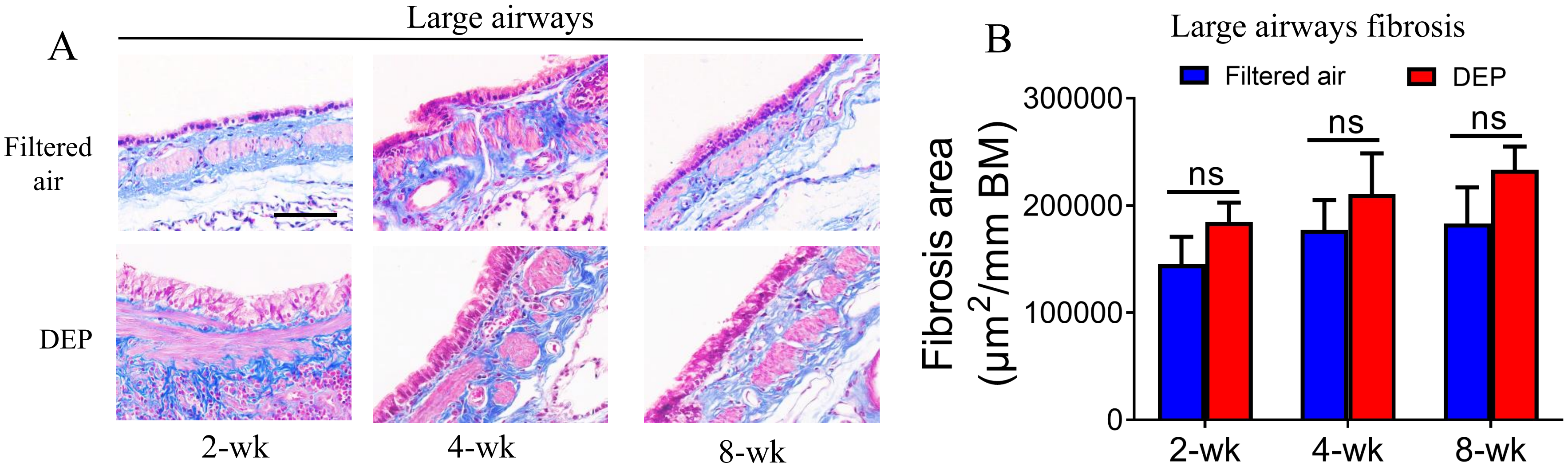

Figure S2. Fibrosis quantification of the large airways. Fibrosis areas around the large airways with perimeter > 2000 µm showed an increasing trend, but did not reach statistical significance following 2wk to 8-wk DEP exposures, respectively. Bar in the Masson Trichrome staining image =100 µm. n=6 rats/group. ns = not significant.

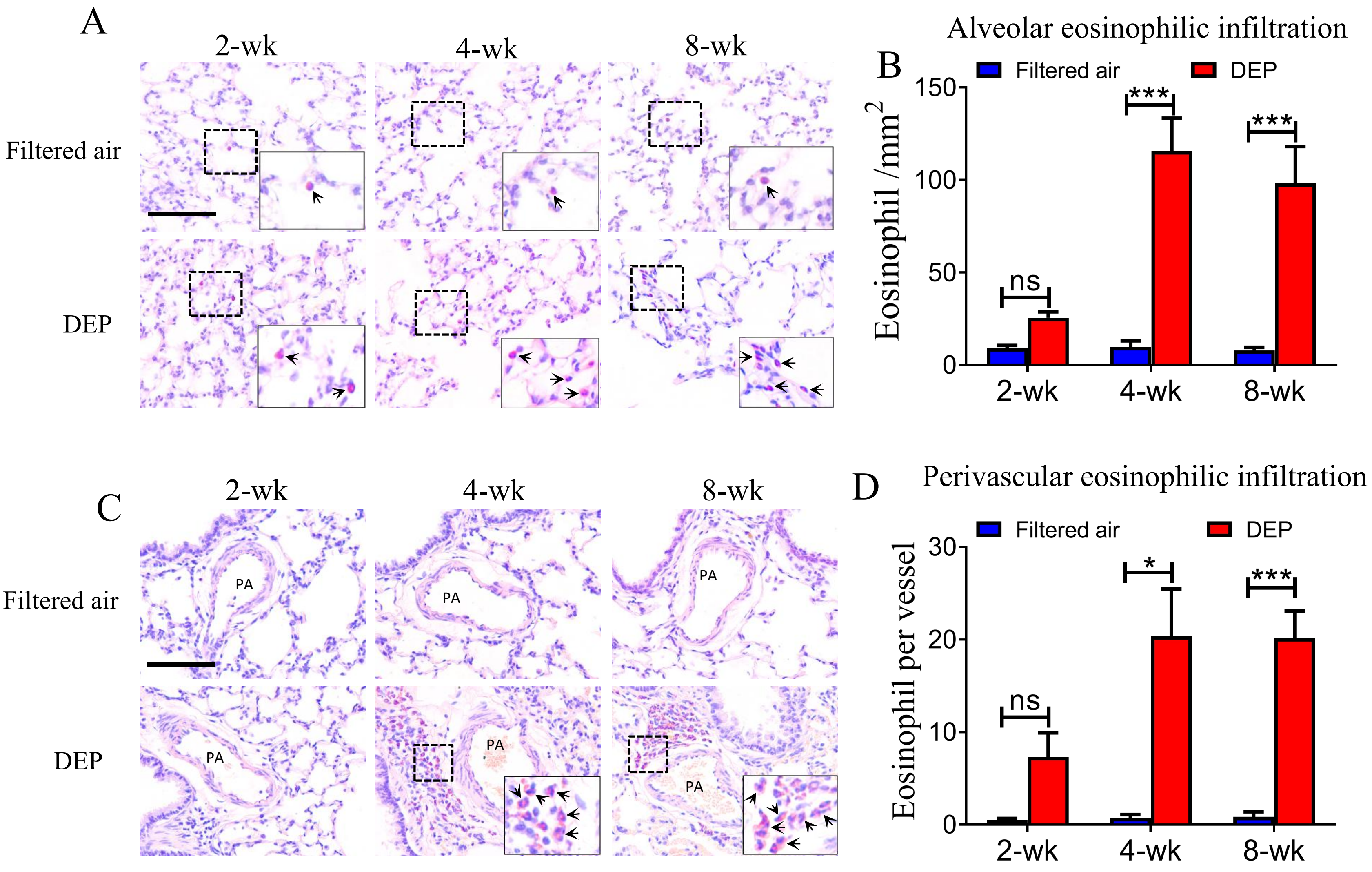

Figure S3. Recruitment of eosinophils (black arrows) to the alveolar region (Panel A) and pulmonary arteries (Panel C). Eosinophil infiltrated in the alveolar increased significantly in rats exposed DEP for 4 weeks and 8 weeks(B) . Rats exposed to DEP for 4 weeks and 8 weeks, but not for shorter durations, demonstrated a significant increase of eosinophils around the PA (D). Bar in the C2R staining image = 100µm. * $p < 0.05$, *** $p < 0.001$. n=6 rats/group. PA = pulmonary arteries.

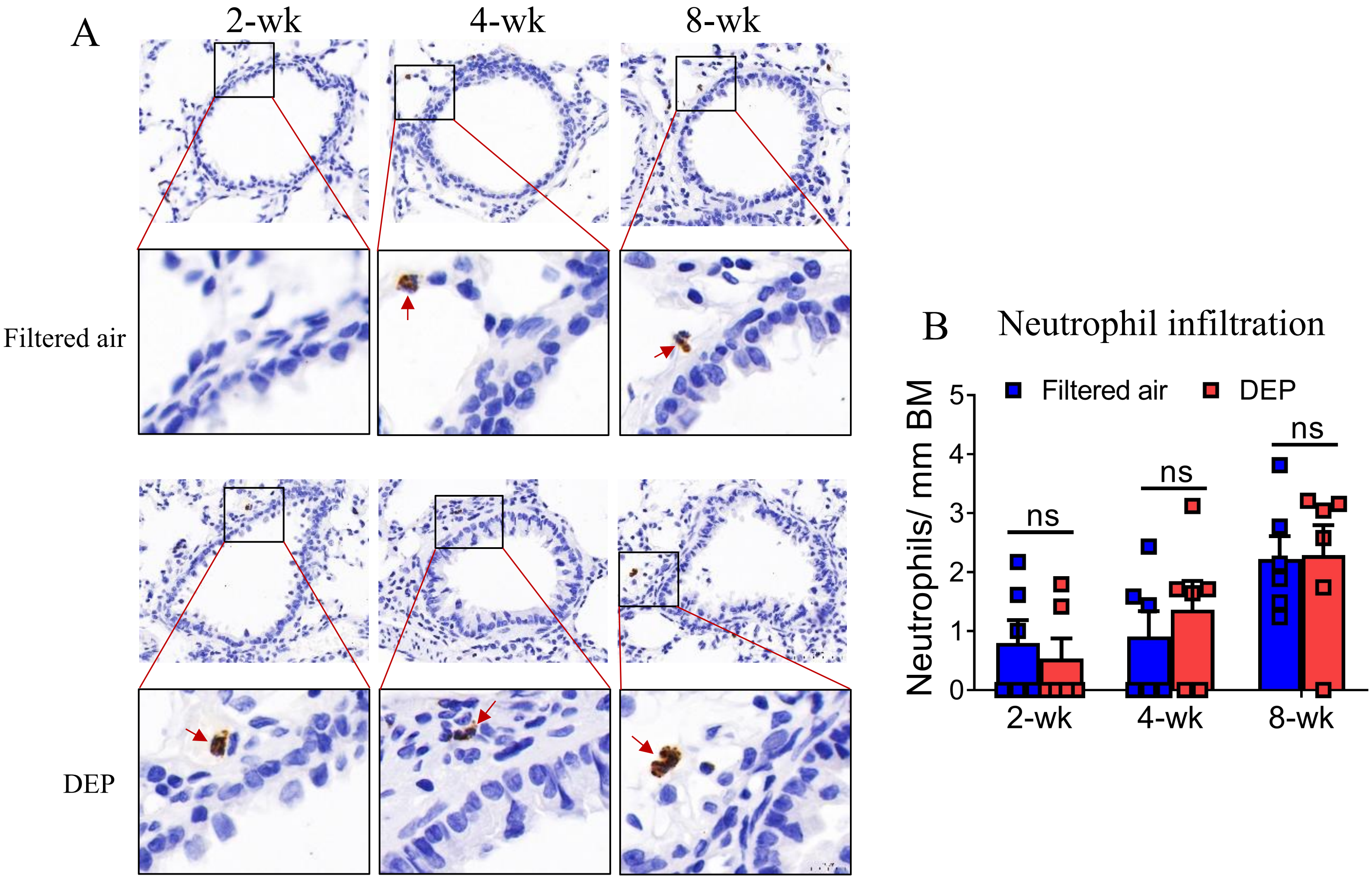

Figure S4. Representative images of IHC staining of neutrophils in small airways, showing the recruitment of neutrophils (red arrows) around the small airways in DEP exposure group and the control group, by exposure duration (Panel A). T-tests showed that neutrophil infiltration of the small airways did not reach statistical difference between the DEP exposure groups and filtered air controls (Panel B). Magnification of IHC staining image, lower panel, 1000x, n= 6 rats/group. ns = not significant.

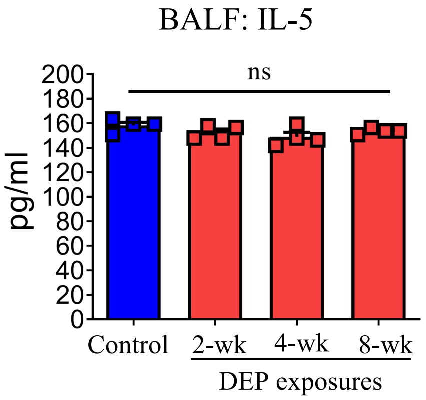

Figure S5. DEP exposures over the courses of 2-wk to 8-wk did not significantly change the concentrations of IL-5 in BALF. ns = not significant.
